# Leptin favors Th17/Treg cell subsets imbalance associated with allergic asthma severity

**DOI:** 10.1002/clt2.12153

**Published:** 2022-06-14

**Authors:** Carolina M. Vollmer, Aleida S. O. Dias, Lana M. Lopes, Taissa M. Kasahara, Letícia Delphim, Júlio Cesar C. Silva, Lucas Paulo Lourenço, Hilary Cesário Gonçalves, Ulisses C. Linhares, Sudhir Gupta, Cleonice A. M. Bento

**Affiliations:** ^1^ Department of Microbiology and Parasitology Federal University of the State of Rio de Janeiro Rio de Janeiro Brazil; ^2^ Post‐graduate Program in Microbiology University of the State of Rio de Janeiro Rio de Janeiro Brazil; ^3^ Pulmonology Service Federal University of the State of Rio de Janeiro Rio de Janeiro Brazil; ^4^ Department of Morphological Sciences Federal University of the State of Rio de Janeiro Rio de Janeiro Brazil; ^5^ Department of Medicine University of California Irvine California USA

**Keywords:** allergic asthma, leptin, obesity, regulatory T cells, Th17 cells

## Abstract

**Background:**

Obesity has often been associated with severe allergic asthma (AA). Here, we analyzed the frequency of different circulating CD4^+^T‐cell subsets from lean, overweight and obese AA patients.

**Methods:**

Mononuclear cells from peripheral blood were obtained from 60 AA patients and the frequency of different CD4^+^T‐cell subsets and type 1 regulatory B cells (Br1) was determined by cytometry. The effect of obese‐related leptin dose on cytokine production and Treg cell function in AA‐derived CD4^+^ T cell cultures was evaluated by ELISA and 3H thymidine uptake, respectively. Leptin levels were quantified in the plasma by ELISA. According to the BMI, patients were stratified as lean, overweight and obese.

**Results:**

AA severity, mainly among obese patients, was associated with an expansion of hybrid Th2/Th17 and Th17‐like cells rather than classic Th2‐like cells. On the other hand, the frequencies of Th1‐like, Br1 cells and regulatory CD4^+^ T‐cell subsets were lower in patients with severe AA. While percentages of the hybrid Th2/Th17 phenotype and Th17‐like cells positively correlated with leptin levels, the frequencies of regulatory CD4^+^ T‐cell subsets and Br1 cells negatively correlated with this adipokine. Interestingly, the obesity‐related leptin dose not only elevated Th2 and Th17 cytokine levels, but also directly reduced the Treg function in CD4^+^ T cell cultures from lean AA patients.

**Conclusion:**

In summary, our results indicated that obesity might increase AA severity by favoring the expansion of Th17‐like and Th2/Th17 cells and decreasing regulatory CD4^+^T cell subsets, being adverse effects probably mediated by leptin overproduction.

## 1 INTRODUCTION

Asthma is a chronic condition of the lower airways classified as allergic and non‐allergic.^1^ This clinical condition can significantly reduce the patient's quality of life, in addition to generating economic impacts on health care systems.^2^, ^3^ Depending on the number of episodes and the therapeutic treatment required to control exacerbations, persistent asthma can be classified as mild, moderate or severe, with the last being potentially fatal due to the irreversibility of bronchial hyperresponsiveness,^4^ even with the standard treatment applying long‐acting β2 agonists (bronchodilators) and oral corticoids.

Asthma is a not a single entity, it comprises of complex disease involving different immune mechanisms (endotypes) and variable clinical presentations (phenotypes). Classically, allergic asthma (AA) is endotype triggered by innocuous environmental substances called allergens and involves the activation of allergen‐specific T helper 2 (Th2) cells and production of immunoglobulin E (IgE).^5^, ^6^ The hallmark of this endotype is the production of high levels of interleukin (IL)‐4, IL‐5 and IL‐13 that favor not only IgE production, but also activation of mast cells and eosinophils in the respiratory tract of AA patients following allergen exposure. Moreover, IL‐5 and IL‐13 produced by local group 2 innate lymphoid cells (ILC‐2) in response to epithelial cell‐derived IL‐25, IL‐33 and thymic stromal lymphopoietin protein (TSLP) amplify Th2‐mediated AA.^6^, ^7^ In this endotype, the symptoms are particularly a result of biological actions of newly synthetized sulphidopeptide leukotrienes (LTC4, LTD4, and LTE4) and platelet‐activating factor (PAF),^8^ mainly produced by activated eosinophils. These pro‐inflammatory lipids play a pivotal role in the pathogenesis of acute attacks due to their ability to provoke local vasodilatation, edema formation, local neurogenic stimulation, smooth muscle contraction and mucus hypersecretion. However, as aforementioned, the pathogenesis of asthma is even more complex, and some patients, particularly those with resistance to inhaled corticosteroids, present an endotype non‐T2 of the disease characterized by intense infiltration of neutrophils in the respiratory airways during exacerbation,^1^, ^4^, ^9^ suggesting the involvement of Th17 cells in severe forms of asthma.^10^

In humans, pathogenic Th17 differentiation appears to be induced by IL‐23^high^DCs,^11^ with increased levels of both IL‐17 and IL‐23 being found in the serum and lungs of patients with severe asthma.^10^, ^12^, ^13^, ^14^, ^15^ Moreover, the mixed‐granulocytic endotype, characterized by elevated levels of eosinophils and neutrophils in bronchial‐alveolar lavages, involves the induction of dual IL‐17 and IL‐4‐secreting CD4^+^ T‐cells in some patients with severe asthma.^16^, ^17^ Interestingly, in vitro studies have indeed revealed a greater sensitivity of Th2 cells to glucocorticoids when compared to Th17 and IL‐4^+^ Th17 cells.^18^ Furthermore, in addition to effector CD4^+^ T cell subsets, asthma severity should also be associated with functional impairment of regulatory lymphocyte compartment capable of producing IL‐10, such as T cells that express (Tregs), or not (Tr1), the FoxP3 marker, as well as type 1 regulatory B cells (Br1).^19^, ^20^, ^21^ The existence of several endotypes of the disease with different responses to therapy might be associated with a complex and poorly understood relationship between genetic factors and environmental events, such as obesity.

Obesity can be defined as a chronic disease mediated by abnormal and excessive accumulation of fat in the body, leading to adverse effects on physical, social and mental wellbeing.^22^ The prevalence of both asthma^23^ and obesity^24^ has increased over the past 20 years, and prospective epidemiological meta‐analysis studies indicate that asthma and obesity coexist in many patients.^25^ In these patients, obesity is an important risk factor for greater frequency and severity of asthma exacerbation and poor response to therapy.^26^ This adverse association may involve increased production of different adipokines, such as leptin.

In addition to the increased frequency of both pro‐inflammatory M1 macrophages and effector CD4^+^ and CD8^+^ T‐cells in the adipose tissue, visceral obesity is normally characterized by hyperleptinemia.^27^ Apart from its role in regulating balance energy expenditure and nutritional status, leptin is also critical for normal T cell response.^28^ Nonetheless, elevated leptin levels have been associated with hypersensitive reactions mediated by both Th2^29^ and Th17^30^ phenotypes. Moreover, by inducing tumor necrosis factor‐*α* (TNF‐α) and IL‐6 production, obesity‐associated leptin levels also damage Treg function, which reduces the production of anti‐inflammatory cytokines such as IL‐10.^31^, ^32^ In asthmatic patients, high leptin levels were inversely correlated with lung function in asthmatic patients.^32^ In a recent study published by our group,^30^ a direct correlation between AA severity with both plasma leptin concentrations and levels of IL‐5, IL‐6 and IL‐17, released by purified CD4^+^ T‐cells, was observed. Nonetheless, this study did not analyze the relationship between different CD4^+^ T cell phenotypes and AA severity. Therefore, the objective of the present study was to characterize the proportions of different effector and regulatory CD4^+^ T cell subsets, as well as Br1, according to AA severity. Also, the direct effect of obese‐related leptin dose on the cytokine production by CD4^+^ T cells was additionally investigated. It is possible that immune imbalance associated with obesity and elevated leptin levels favor the expansion of different CD4^+^ T cell subsets associated with AA severity.

## 2 MATERIAL AND METHODS

### 2.1 Subjects

Sixty patients with AA (47 women and 13 men) were recruited from March 2017 to November 2019 from the Federal University of the State of Rio de Janeiro Hospital/UNIRIO (Rio de Janeiro, Brazil). Persistent asthma was diagnosed by a history of recurrent wheezing, dyspnea and chest tightness, and confirmed by methacoline bronchial hyperresponsiveness [mild (PC20 ≥ 1 but ≤4 mg/ml) or moderate to severe (PC20 < 1 mg/ml)], when FEV1 was ≥70%, or bronchial reversibility after salbutamol inhalation (when FEV1 was <70%). According to the Global Initiative for Asthma criteria,^5^ AA patients were subdivided into 3 groups: mild [*n* = 20, FEV1 (% predicted) 81–101; FEV1 reversibility (%) 11–18], moderate [*n* = 20, FEV1 (% predicted) 61–85; FEV1 reversibility (%) 3.9–14] and severe [*n* = 20, FEV1 (% predicted) 39–77; FEV1 reversibility (%) 2.6–17]. Patients were allowed to receive treatment with inhaled corticosteroids for 2 months prior to the study, but not with systemic steroids. All patients had a positive skin prick test, defined as a >5‐mm diameter skin wheal response to at least 1 of 6 common allergens (*Dermatophagoides pteronyssinus, D. farinae, Alternaria,* mixed grass pollen, dog and cat hair). The great majority of patients are polysensitized (15% of patients had positive reaction to 1 or 2 allergens and 85% had a positive reaction to 3 or more allergens). The presence of rhinitis was observed in 65% (*n* = 39) and the severity of symptoms was determined by using total nasal symptom score (TNSS) (sneezing, congestion, itching, and rhinorrhea)^33^ (Table 1). Of note, relevant clinical allergens were recorded to dust mites (*n* = 31), cat dander (*n* = 12), dust mites and cat dander (*n* = 13), *Alternaria* (*n* = 2) and mixed grass pollen (*n* = 2). The occurrence of infectious or other autoimmune diseases were excluded by clinical and serological tests. Twenty healthy subjects (15 women and 5 men), matched by age, gender and body mass index (BMI) were also recruited for the control group. BMI is calculated from the mass (weight in Kg) and height (in meters) of an individual adopting the formula (BMI = kg/m^2^). Subjects were stratified as lean (BMI from 18.5 to 24.9), overweight (BMI from 25 to 29.9) and obese class I (BMI from 30 to 35) according to BMI. In the present study, all subjects included were nonsmokers, with no history of upper or lower airway infectious diseases 4 months prior to recruitment in the study. We also excluded individuals taking oral or intravenous steroids, theophylline, long‐acting β2‐agonists, leukotriene antagonists or antihistamines 2 months prior to the study. The Ethics Committee for Research on Human Subjects at the Federal University of the State of Rio de Janeiro (UNIRIO) approved the study, and blood was collected only after written informed consent was obtained from each individual.

**TABLE 1:** Subject characteristics

		AA^b^		
	Control^a^	Mild	Moderate	Severe
No. of subjects (n)	20	20	20	20
Gender (female/male) (n)	15/5	14/6	16/4	17/3
Age [(years), mean ± SD]	31.9 ± 13.1	40.7 ± 18.1	40.2 ± 19.7	42.9 ± 16.8
BMI (*n*)^c^				
*Lean*	7	7	6	7
*Overweight*	6	6	6	5
*Obese class I*	7	7	8	8
Rhinitis, symptoms severity, *n* ^d^				
*None*	0	7	4	6
*Mild*	0	9	2	1
*Moderate*	0	3	5	8
*Severe*	0	1	9	5

^a^ Healthy individuals.

^b^ Patients with mild (*n* = 20), moderate (*n* = 20) and severe (*n* = 20) allergic asthma (AA).

^c^ Body mass index: a value derived from the mass (weight in Kg) and height (in meters) of an individual (lean: 18.5–24.9, overweight: 25–29.9 and obese class I: 30–35).

^d^ TNSS,^34^ total nasal symptom score [Rhinorrhea, nasal itching, nasal obstruction, and sneezing [scored as 0 (none), 3–6 points (mild), 7–9 points (moderate), and 10–12 points (severe)].

### 2.2 Flow cytometry analysis

Peripheral blood was collected in heparin‐containing tubes (BD Vacutainer, Franklin Lakes, NY) and peripheral blood mononuclear cells (PBMC) were obtained by centrifugation on the Ficoll–Hypaque density gradient. Fresh viable PBMC (1 × 10^6^/ml) were cultured in 24‐well flat‐bottomed microplates with 2 ml of RPMI medium (ThermoFisher Scientific Inc.) supplemented with 2 μM of L‐glutamine (GIBCO, Carlsbad, CA, USA), 10% of fetal calf serum, 20 U/mL of penicillin, 20 μg/ml of streptomycin and 20 mM of HEPES buffer. The cells were stimulated with phorbolmyristate acetate (PMA, 20 ng/ml; Sigma‐Aldrich) plus Ionomycin (600 ng/ml; SigmaAldrich) for 4 h in the presence of brefeldin A (10 μg/ml) (BD Biosciences, San Diego, CA, USA). The cell cultures were maintained at 37°C in a humidified 5% CO2 incubator. After 4 h, different CD4^+^ T cell subsets and Br1 cells were identified by staining the PBMC with mouse anti‐human monoclonal antibodies (mAbs) for CD4‐FITC, CD19‐FITC, CD39‐PE‐Cy7, IL‐4‐APC, IL‐10‐APC, IL‐17‐PE‐Cy7, IFN‐γ‐PE, and FoxP3‐PE. These mAbs and all isotype control antibodies were purchased from BD Biosciences (San Diego, CA, USA). Briefly, whole blood cells were incubated with various combinations of mAbs for surface markers (CD4, CD19 and CD39), for 30 min at room temperature in the dark, according to manufacturer's instructions. The cells were washed with PBS +2%FBS, then the red blood cells were lysed with Fix/Lyse solution (eBiosciences) for 10 min at room temperature before cell permeabilization, which was performed by incubating cells with Cytofix/Cytoperm solution (BD Pharmigen, San Diego, CA) at 4°C for 20 min. After washing, the mAbs for intracellular staining (IL‐4‐APC, IL‐17‐PE‐Cy7, IFN‐γ‐PE, IL‐10‐APC, and FoxP3‐PE) were added in different combinations and incubated for 30 min at 4°C. The cells were acquired on Accuri C6 (Accuri™, Ann Arbor, MI, USA) or Attune NxT flow cytometers (Thermo Fisher Corporation) and analyzed using Cflow (Accuri™, Ann Arbor, MI, USA). Isotype control antibodies and single‐stained samples were used to periodically check the settings and gates on the flow cytometer. After acquisition of 200,000–300,000 events, lymphocytes were gated based on forward and side scatter properties after the exclusion of dead cells, by using propidium iodide, and doublets. Additionally, gated cells were negative for CD14 marker.

### 2.3 Leptin quantification

Circulating leptin levels were measured using a commercial ELISA kit following manufacturer's instructions (Enzo Life Sciences, Farmingdale, NY). Plates were read at 450 nm in ELISA reader (Dynex Technologies, USA). Lyophilized leptin ranging from 31.3 to 2000 pg/ml was used to construct the standard curve.

### 2.4 The effect of leptin on cytokine production by CD4^+^ T cells

The CD4^+^ T cells were obtained from PBMC via negative selection using magnetic columns according to manufacturer's instructions (EasySep^TM^, StemCell Technology, Canada). Briefly, 50 μL of the isolation cocktail was added to a cell suspension of 1 × 10^7^ cells in 1 ml of HBSS in a 15 ml tube. After 10 min incubation at room temperature, a volume of 100 μL for CD4 of the RapidSpheres suspensions were added to the cell suspension, followed by further incubation at room temperature for 5 min. Subsequently, 4 ml of HBSS were added to the cell suspension and the tube was then placed on a magnet for 5 min. Finally, the supernatants were recovered. The purity of CD4^+^ T cells was >98%, as measured by flow cytometry (data not shown). The CD4^+^ T cells (1 × 10^5^/ml) were suspended in RPMI‐1640 medium supplemented with 2 μM of L‐glutamine (GIBCO, Carlsbad, CA, USA), 10% fetal calf serum, 20U/ml of penicillin, 20 μg/ml of streptomycin and 20 mM of HEPES buffer, cultured in 24‐well flat‐bottomed microplates in the presence or absence of anti‐CD3/anti‐CD28 beads (10 μL/ml) for 3 days, which corresponds to the period of maximal T cell activation by polyclonal stimuli. In some cultures, an obesity‐related leptin dose (Lep; 50 ng/ml; Figure S1) (Sigma Chemicals, St Louis, MO) was added. After 3 days, the supernatant from different T cell cultures was collected and the cytokines were quantified by ELISA technique using OptEIA ELISA kits (BD, Pharmigen, San Diego, CA), according to manufacturer's instructions. Each ELISA was performed using pairs of antibodies against IL‐4, IL‐5, IL‐13, IFN‐γ, IL‐6, IL‐17A (IL‐17), and IL‐10. The reaction was revealed with streptavidin‐horseradish peroxidase, using 3,3′,5,5′‐tetramethylbenzidine (TMB) as a substrate. Recombinant human cytokines, at concentrations ranging from 3.5 to 500 pg/ml, were used to construct standard curves.

### 2.5 The role of leptin in modulating Treg function

In another set of experiments, following manufacturer's instructions, PBMC cultures were enriched with CD4^+^ CD127^low^ CD25^+^ regulatory T cells (Tregs) and CD4^+^CD25^‐^ responder T cells (Tresp) by using EasySep™ kits (EasySep^TM^, StemCell Technology, Canada). As determined by cytometry, the purity of the Tregs and Tresp cells was >91.4% and >93.9%, respectively (data not shown). For the suppression assay, CD4^+^CD25^+^CD127^neg/low^ T cells (Tregs, 1 × 10^5^/ml) from AA patients were maintained for 24 h in the presence of medium alone or leptin (50 ng/ml). At the end of the incubation period, the cells were washed with medium (RPMI at 5% CFS), to remove residual adipokine, and then co‐cultured with autologous CD4^+^CD25^−^CD127^+^ Tresp at 1:8 and 1:4 Treg/Tresp ratios. Co‐cultures were set up in triplicate, incubated for 3 days at 37°C in the presence of anti‐CD3/anti‐CD28 beads, and cell proliferation was evaluated through 3H thymidine uptake after addition of 1 μCi/well 8 h to the cell cultures before the end of the incubation period. The cells were harvested in glass fiber filters in an automatic cell harvester and radioactive incorporation was measured using a liquid‐scintillation counter.

### 2.6 Statistical analyzes

All statistical analyzes were conducted using the Prism 8.0 program (GraphPad Software). Immunological evaluations were performed in triplicate or quadruplicate for each individual with the intra‐assay variability ranging from 8.9% to 13.7% (median value of 11.2%) as calculated by the software. Comparisons between immune assays in cell cultures from the different groups were performed with ANOVA followed by Tukey test for data with Gaussian distribution and by Kruskal‐Wallis followed by Dunn's test for data without Gaussian distribution. The results were also corrected by Bonferroni. The analysis of correlations between plasma leptin levels and different lymphocyte subtypes was conducted using Spearman correlation. Significance in all experiments was defined as *p* < 0.05.

## 3 RESULTS

### 3.1 Impact of clinical status and obesity on different effector CD4^+^ T‐cell subsets in AA patients

**FIGURE 1:**
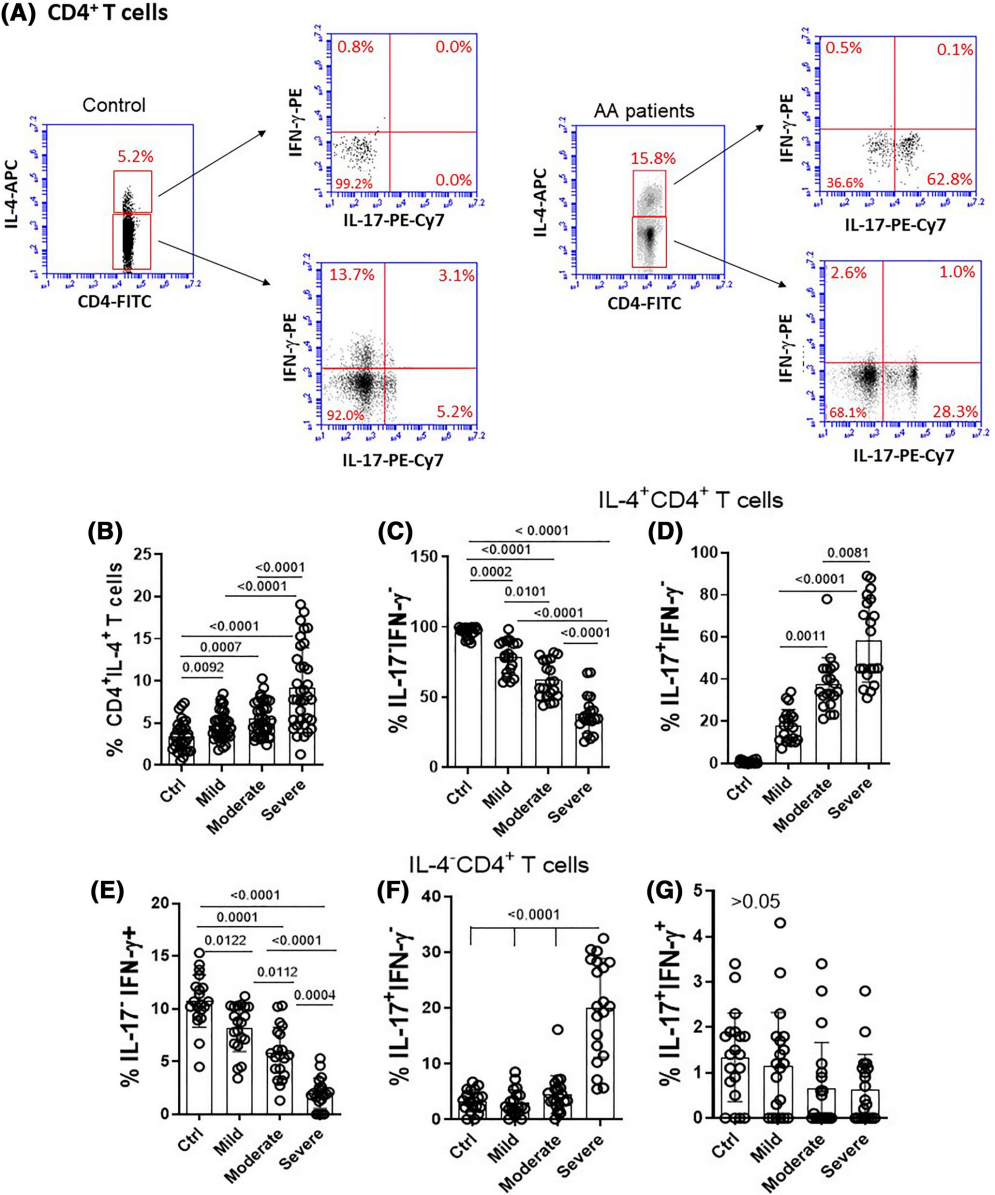
Frequency of circulating CD4^+^ T cell subtypes capable of producing IL‐4, IL‐17 and IFN‐γ in AA patients according to clinical status. PBMC (1 × 10^6^/ml), obtained from healthy subjects (*n* = 20) and patients with mild (= 20), moderate (*n* = 20), and severe (*n* = 20) AA were stimulated for 4 h with PMA (20 ng/ml) and ionomicina (600 ng/ml). Taking into consideration the gating strategy shown in the panel A, the percentage of (B) all IL‐4^+^CD4^+^T cells, as well as (C) Th2‐like (IL‐4^+^IL‐17^−^IFN‐γ^‐^), (D) hybrid Th2/Th17 phenotype (IL‐4^+^IL‐17^+^IFN‐γ^‐^), (E) Th1‐lie (IL‐4^−^IL‐17^−^IFN‐γ^+^), (F) Th17‐like (IL‐4^−^IL‐17^+^IFN‐γ^‐^) and (G) dual Th1/TH17 phenotype were identified by cytometry. The mean values were compared and analyzed between the groups using the one‐way ANOVA and the *p* value shown in the figure

For our study, 60 patients (47 females and 13 male) with mild (*n* = 20), moderate (*n* = 20) and severe (*n* = 20) allergic asthma (AA) were recruited and subsequently stratified by body mass index (BMI). As expected, most patients also suffered from rhinitis (Table 1). Twenty healthy subjects (15 females and 05 male) were also recruited as control (Table 1). Of note, no difference in the CD4^+^ T cell and B cell counts was observed between healthy subjects and AA patients in the different clinical subgroups. Our primary objective was to evaluate the proportion of total IL‐4^+^CD4^+^ T cells in AA patients. Following the gating strategy shown in Figure 1A, a higher proportion of IL‐4^+^CD4^+^ T‐cells was detected in AA patients when compared to individuals from the control group, as expected (Figure 1B). Additionally, among patients, the highest percentage of these cells was detected in samples from individuals with severe AA (Figure 1B).

Based on the combination of cytokines used to identify classical Th1 (IL‐4^−^IL‐17^−^IFN‐γ^+^), Th2 (IL‐4^+^ IL‐17^−^IFN‐γ^‐^) and Th17 (IL‐4^−^IL‐17^+^IFN‐γ^‐^) phenotypes, we observed that, in the control group, the great majority of total IL‐4^+^CD4^+^ T‐cells expressed neither IL‐17 nor IFN‐γ (Figure 1C). Interestingly, in patients, the severity of the disease was associated with an expansion of IL‐4^+^IL‐17^+^IFN‐γ^‐^ CD4^+^ T‐cells (Figure 1D) rather than classic Th2‐like cells (Figure 1C). This hybrid Th2/Th17 cell subtype was practically absent in the control group (Figure 1D). Among CD4^+^ T cells negative for IL‐4, the proportion of IFN‐γ^+^IL‐17^‐^ (type Th1) was significantly higher in healthy individuals in comparison to patients (Figure 1E). In the AA group, an important decrease in the frequency of Th1 cells was observed in patients with severe AA (Figure 1E). In contrast, a higher frequency of Th17‐like cells was detected in patients with severe AA when compared to all other individuals (Figure 1F). No difference was observed in the proportion of this phenotype between the control group and patients with mild or moderate AA (Figure 1F). Finally, the percentage of IL‐4^−^CD4^+^ T cells capable of co‐expressing IL‐17 and IFN‐γ was low and no difference between the different groups of subjects was observed (Figure 1G).

**FIGURE 2:**
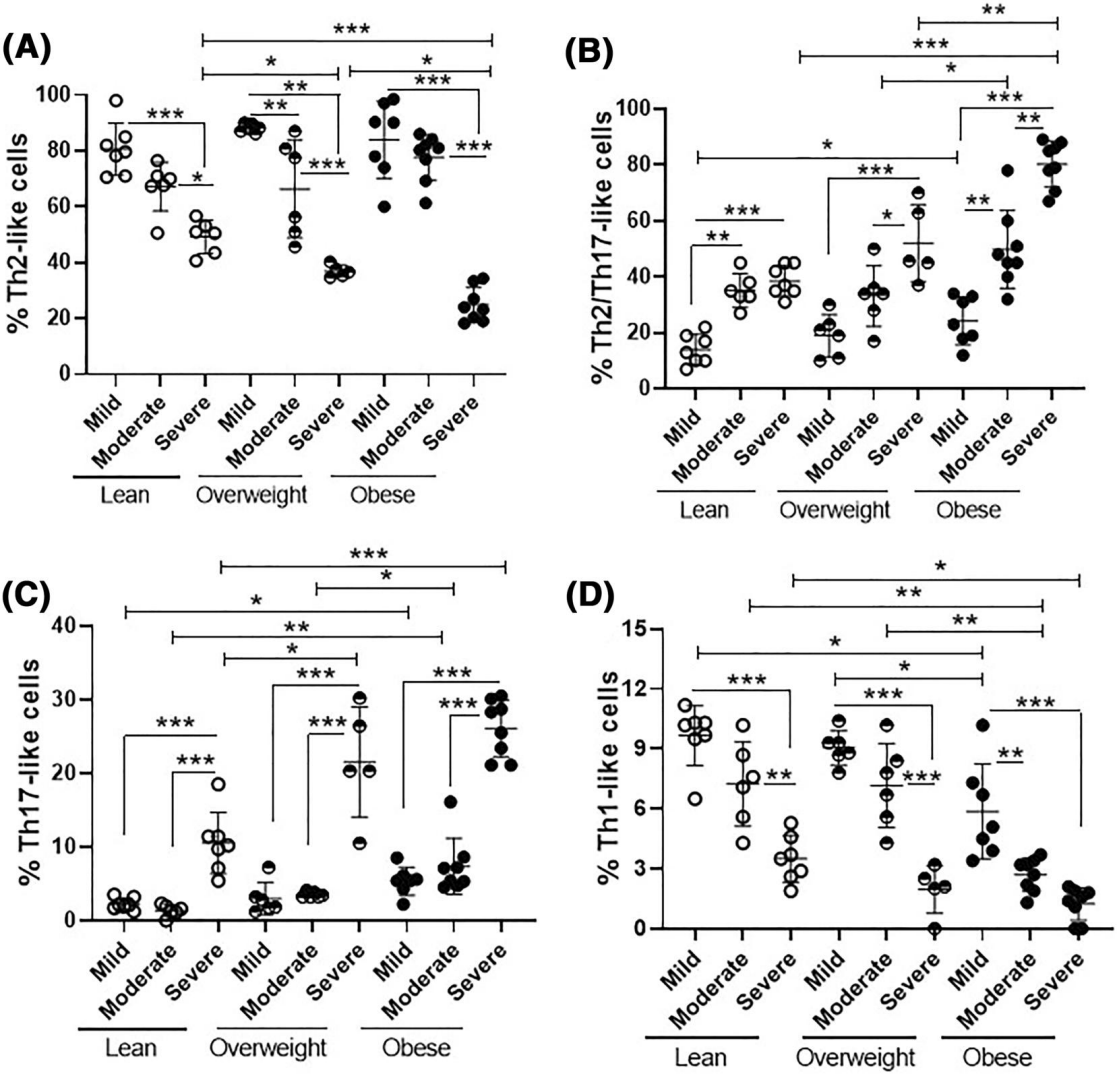
Frequency of different subtypes of circulating CD4^+^ T cells capable of producing IL‐4, IL‐17 and IFN‐γ in AA patients according to BMI. PBMC (1 × 10^6^/ml), obtained from healthy subjects (*n* = 20) and patients with mild (= 20), moderate (*n* = 20) and severe (*n* = 20) AA were stimulated for 4 h with PMA (20 ng/ml) and ionomicina (600 ng/ml), and the percentage of CD4^+^ T cells related to (A) Th2 (IL‐4^+^IL‐17^−^IFN‐γ^‐^), (B) Th2/Th17 (IL‐4^+^IL‐17^+^IFN‐γ^‐^), (C) Th17 (IL‐4^−^IL‐17^+^IFN‐γ^‐^) and (D) Th1 (IL‐4^−^IL‐17^−^IFN‐γ^+^) phenotypes from patients were stratified by BMI within each clinical subgroup of AA (mild, moderate or severe). The mean values were compared and analyzed between the different groups using the two‐way ANOVA and (*), (**), (***) indicate *p* < 0.05, <0.001 and <0.0001

Regarding the BMI of patients, although the percentage of total CD4^+^ T cells had not been associated with fat mass, the frequency of Th2‐like cells was significantly lower in obese individuals with severe disease when compared to mild and moderate AA (Figure 2A). No significant difference was observed in the groups with mild and moderate AA in terms of Th2 cells (Figure 2A). On the other hand, a higher percentage of the hybrid Th2/Th17 phenotype was observed in obese patients with severe AA, with no significant difference between patients with mild or moderate forms of the disease (Figure 2B). Similarly, the highest percentages of Th17‐like cells were observed in obese AA patients, especially those with severe disease (Figure 2C). In contrast, both the severity and excessive weight gain negatively impacted the frequency of Th1‐like cells in AA patients (Figure 2D).

### 3.2 Obesity amplifies damage in the regulatory lymphocyte compartment in patients with AA

**FIGURE 3:**
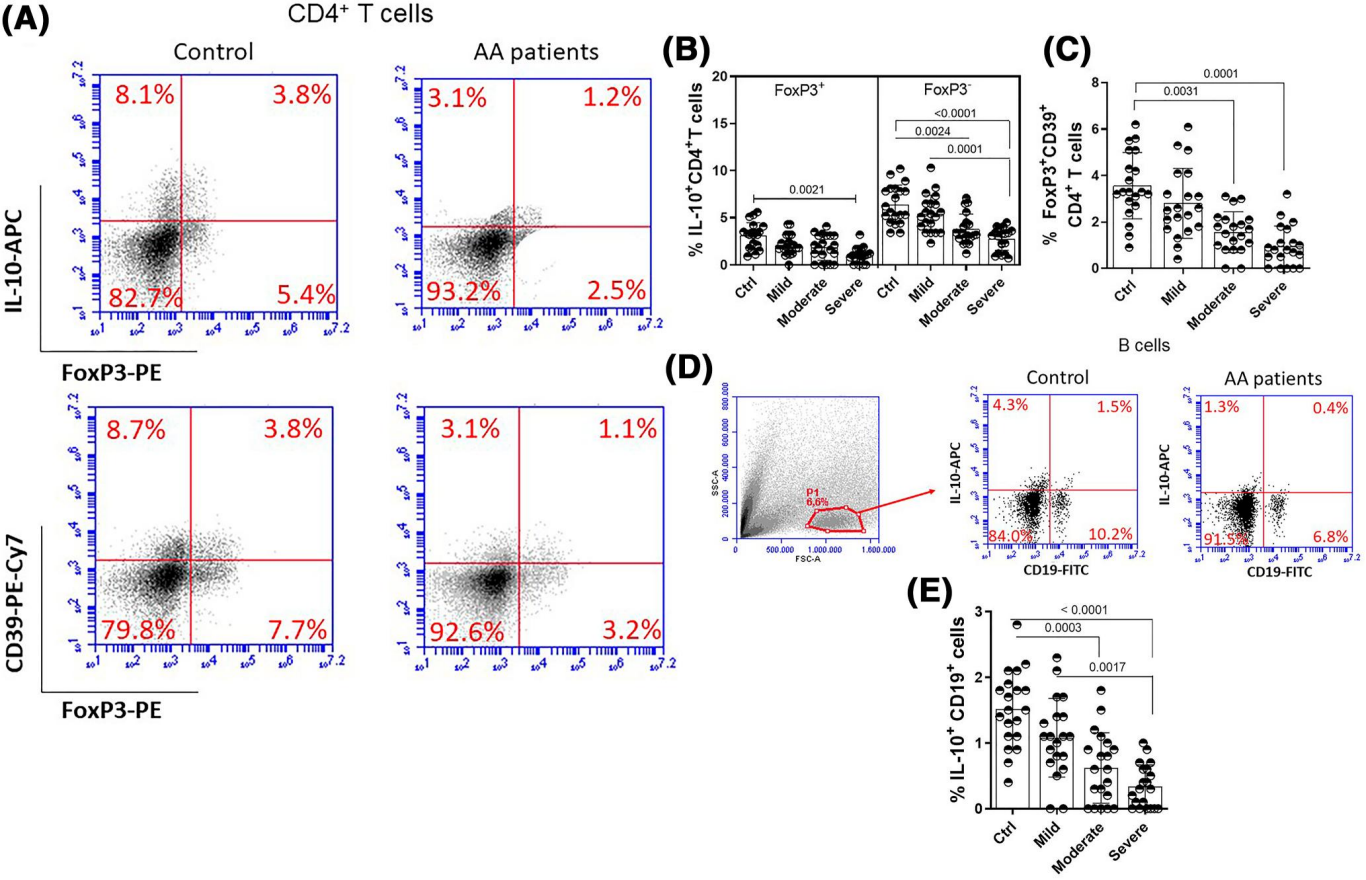
Frequency of regulatory CD4^+^ T cells and Br1 cells in AA patients according to clinical status. PBMC (1 × 10^6^/ml), obtained from healthy subjects (*n* = 20) and patients with mild (= 20), moderate (*n* = 20) and severe (*n* = 20) AA were stimulated for 4 h with PMA (20 ng/ml) and ionomicina (600 ng/ml), and, following the gating strategy shown in the panel A, the percentage of IL‐10^+^ CD4^+^ T cells able to express or not FoxP3 protein (B) as well as FoxP3^+^CD39^+^ CD4^+^ T cells (C) was evaluated by cytometry. In (D) and (E), the identification strategy and mean values of regulatory B cells (CD19 + IL‐10 ^+^) are shown, respectively. The mean values were compared and analyzed between the groups using the one‐way ANOVA and the *p* value shown in the figure

Following the gating strategy for identifying CD4^+^ T cells that express the FoxP3, IL‐10 and CD39 markers demonstrated in Figure 3A, we observed a lower frequency of IL‐10^+^Foxp3^+^CD4^+^ T cells (Tregs) in patients with severe AA when compared to the control group. Among IL‐10^+^FoxP3^−^CD4^+^ T cells, known as Tr1 cells,^34^ a lower percentage was observed in patients with moderate and severe AA when compared to the control group (Figure 3B), without statistical difference between healthy individuals and mild AA patients. Amongst the patients, the lowest percentage of Tr1 cells was observed among those with severe AA (Figure 3B). The acquisition of the CD39 marker has been associated with high functional capacity of FoxP3^+^ CD4^+^ T‐cells.^35^ Figure 3C shows that the percentage of these cells was significantly higher in healthy individuals when compared with patients with moderate and severe AA, but not when compared to those with mild AA. Amongst patients, the frequency of this subtype of regulatory T cell was highly variable with no significant difference according to AA severity (Figure 3C). As for Br1 cells, identified as IL‐10^+^CD19^+^ cells (Figure 3D), a lower percentage was identified in the samples obtained from patients with moderate and severe AA when compared to the control group (Figure 3E). Among patients, the frequency of Br1 cells was significantly higher in those with mild AA compared to individuals with severe forms of the disease (Figure 3E).

**FIGURE 4:**
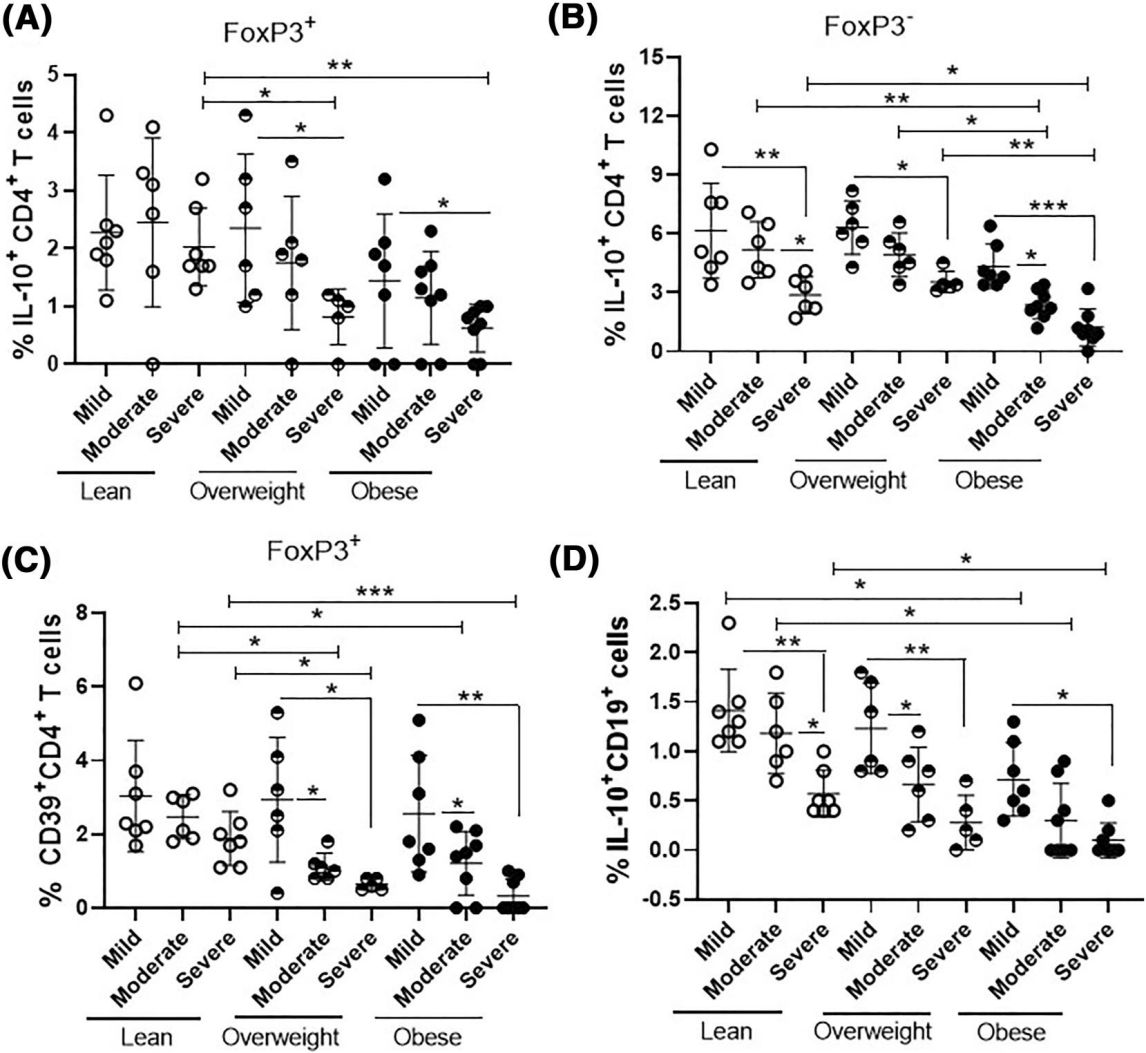
Frequency of CD4^+^ T lymphocyte subtypes and Br1 cells in AA patients according to BMI. The proportion of IL‐10^+^CD4^+^ T cells capable (A) or not (C) of expressing FoxP3, as well as (B) FoxP3^+^CD39^+^CD4^+^ T cells and (D) IL‐10^+^B lymphocytes was stratified by BMI for each clinical group of patients with mild, moderate or severe AA. The percentage of different cell types was evaluated after stimulating PBMC (1 × 10^6^/ml) for 4 h with PMA (20 ng/ml) and ionomicina (600 ng/ml) by cytometry using anti‐CD4, anti‐CD39, anti‐CD19, anti‐FoxP3 and anti‐IL‐10. The mean values were compared and analyzed between the groups using the two‐way ANOVA and (*), (**), (***) indicate *p* < 0.05, <0.001 and <0.0001

Regarding BMI, obesity was associated with a lower percentage of IL‐10^+^FoxP3^+^CD4^+^ T‐cells only in patients with severe AA when compared to other patients and the control group (Figure 4A). In addition, the percentage of CD39^+^FoxP3^+^CD4^+^ T‐cells was significantly lower in overweight or obese patients who presented moderate or severe forms of AA (Figure 4C). As for Tr1 cells, a lower frequency of this cell subtype was observed only in obese patients with moderate and, mainly, severe AA (Figure 4B). As demonstrated in Figure 4D, the presence of obesity was also associated with a lower percentage of IL‐10^+^CD19^+^ cells among patients with mild, moderate and, mainly, severe AA.

### 3.3 Elevated plasma leptin levels are related to an imbalance of CD4^+^ T‐cell subsets and Br1 cells implicated in AA severity

**FIGURE 5:**
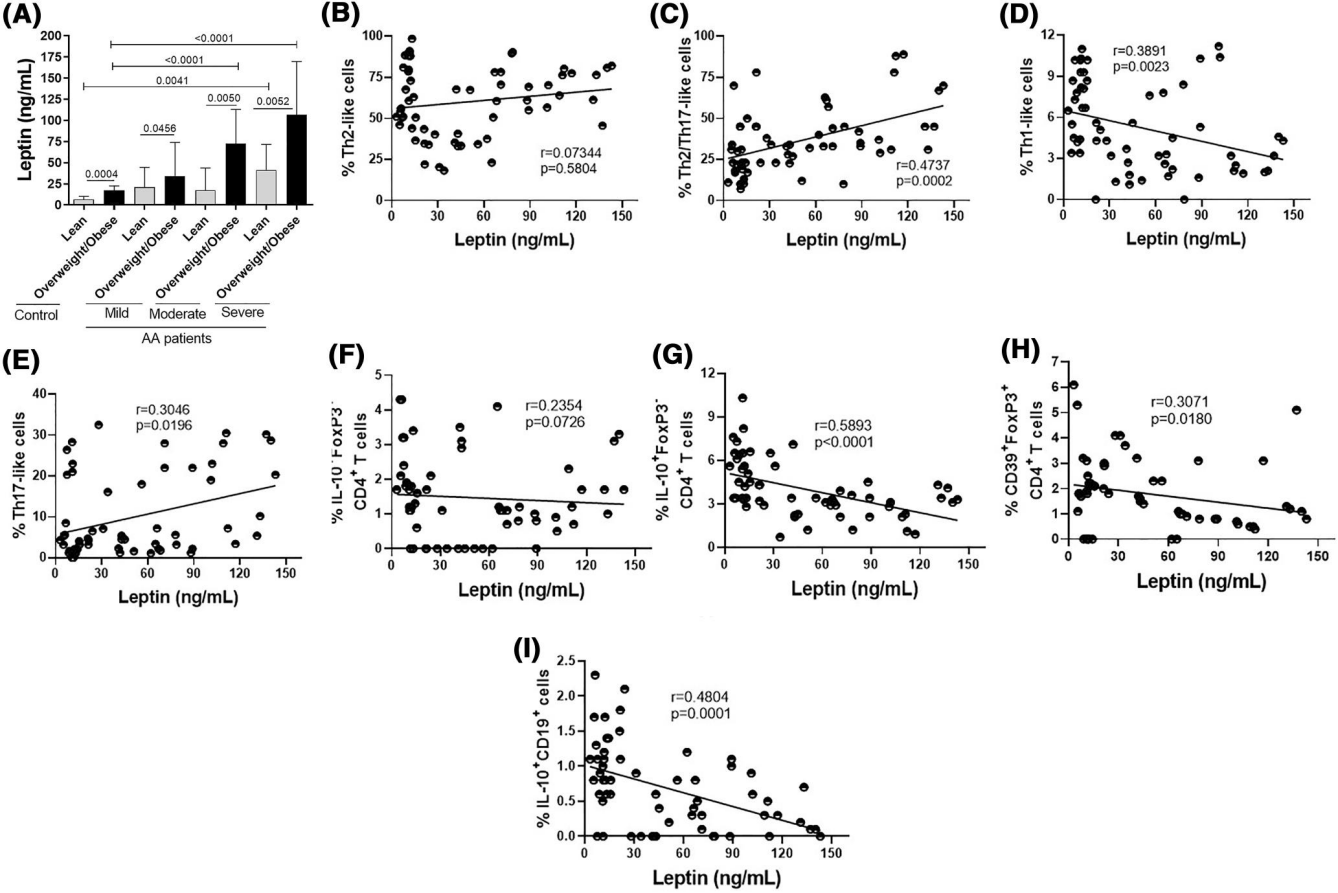
Plasma leptin dosage and its correlation with the frequency of different CD4^+^ T cell subtypes and circulating Br1 cells in AA patients. In (A), plasma from healthy subjects (*n* = 20) and patients with mild (*n* = 20), moderate (*n* = 20) and severe (*n* = 20) AA with different BMI values, were submitted to leptin dosage by ELISA and mean values were compared between the different groups. In (B‐E), the leptin levels were correlated with the percentage of different CD4^+^ T cell subsets related with (B) Th2 (IL‐4^+^IL‐17^‐^ IFN‐γ^‐^), (C) Th2/Th17 (IL‐4^+^IL‐17^+^IFN‐γ^‐^), (D) Th1 (IL‐4^−^IL‐17^‐^ IFN‐γ^+^), and (E) Th17 (IL‐4^−^IL‐17^+^IFN‐γ^‐^) phenotypes, as well as IL‐10^+^ CD4^+^ T cells, expressing (F) or not FoxP3 (G), CD39^+^FoxP3^+^CD4^+^ T cells (H) and Br1 cells (I). The variables were submitted to Spearman's correlation and the *p* values indicated in the figure

Many of the immune disorders in obese patients have been associated with high production of certain adipokines, particularly leptin.^28^, ^29^, ^30^, ^31^, ^32^, ^36^, ^37^ As expected, plasma leptin levels were significantly lower in lean subjects from the control group and AA patients when compared to their overweight/obese counterparts (Figure 5A). However, and interestingly, among lean subjects, leptin concentrations were higher in severe AA patients (Figure 5A). Similarly, among the overweight/obese subjects, circulating levels of this adipokine were also significantly higher in AA patients with moderate and severe disease (Figure 5A). Regarding the different CD4^+^ T‐cell phenotypes, a positive and significant correlation was observed between plasma leptin levels and the percentages of hybrid Th2/Th17 phenotype (Figure 5C) and Th17‐like cells (Figure 5E). On the other hand, an inverse and significant correlation was observed between this adipokine and the proportion of Th1‐type cells (Figure 5D). No correlation was observed between plasma leptin concentrations and the percentage of Th2‐type cells (Figure 5B). With regard to regulatory phenotypes, a significant decrease in the percentage of Tr1 cells (Figure 5G) and CD39^+^FoxP3^+^CD4^+^ T‐cells (Figure 5H), as well as Br1 cells (Figure 5I), was observed in patients with higher circulating levels of this adipokine in patients with AA, with no significant difference in the proportion of IL‐10^+^FoxP3^+^CD4^+^ T‐cells (Figure 5F). In the control group, similar results were observed. While leptin levels positively correlated with the percentage of Th17‐like cells, a negative correlation was observed between this adipokine and the frequency CD39^+^FoxP3^+^CD4^+^ T‐cells and Tr1 cells (Figure S2).

### 3.4 Effect of leptin on in vitro cytokine production and Treg function in CD4^+^ T cells from AA patients

**FIGURE 6:**
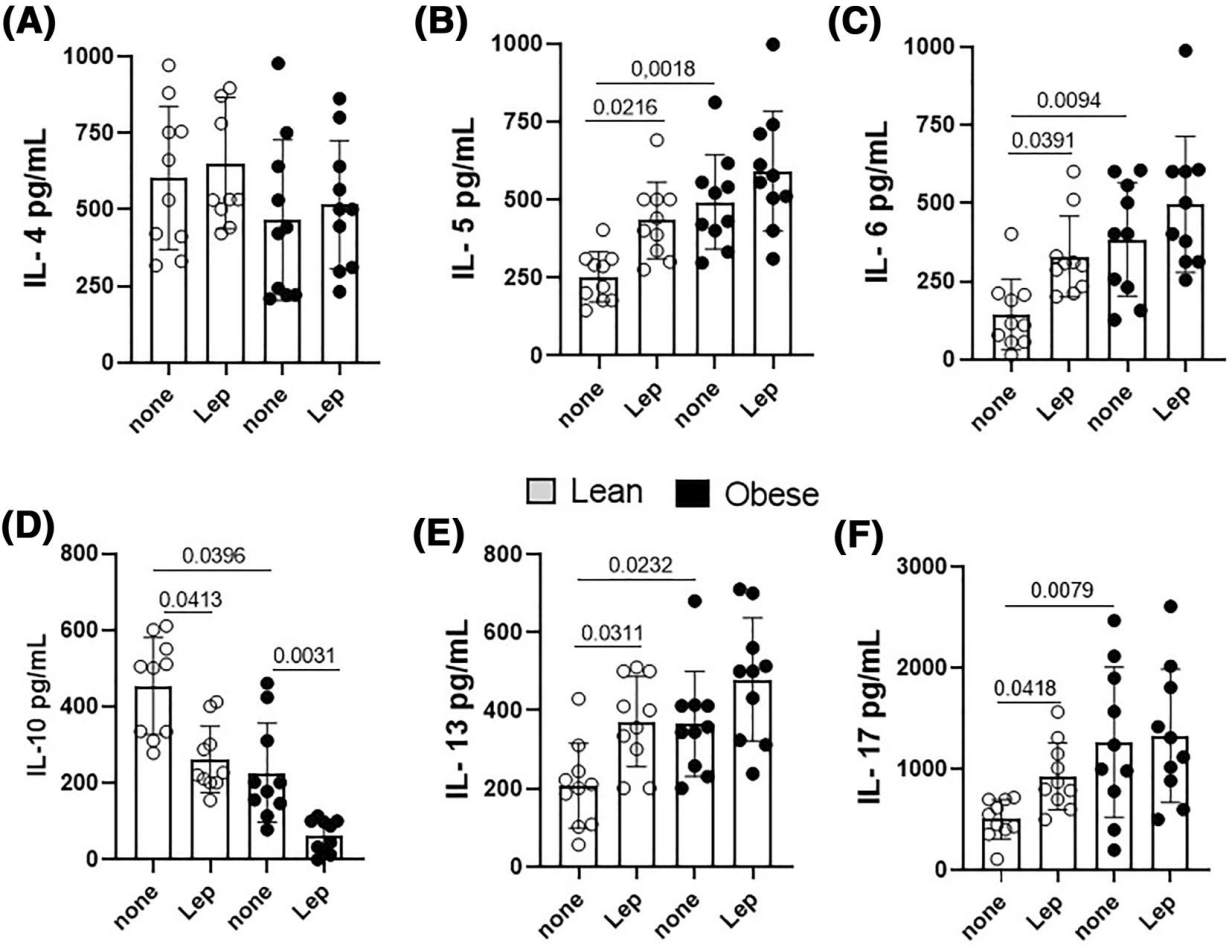
The role of leptin in cytokine production by CD4^+^ T cells from AA patients. CD4^+^ T cells (1 × 10^6^/ml) from lean (*n* = 10) and obese (*n* = 10) AA patients were stimulated with anti‐CD3/anti‐CD28 beads (10 μL/ml) in the presence of leptin (50 ng/ml). After 3 days, the supernatants were collected and the cytokine contents assayed by ELISA. Data are shown as mean ± SD of 5 independent experiments with 1 lean and 1 obese AA sample per experiment. Significance was calculated by comparing the different groups and the *p* values indicated in the figure

**FIGURE 7:**
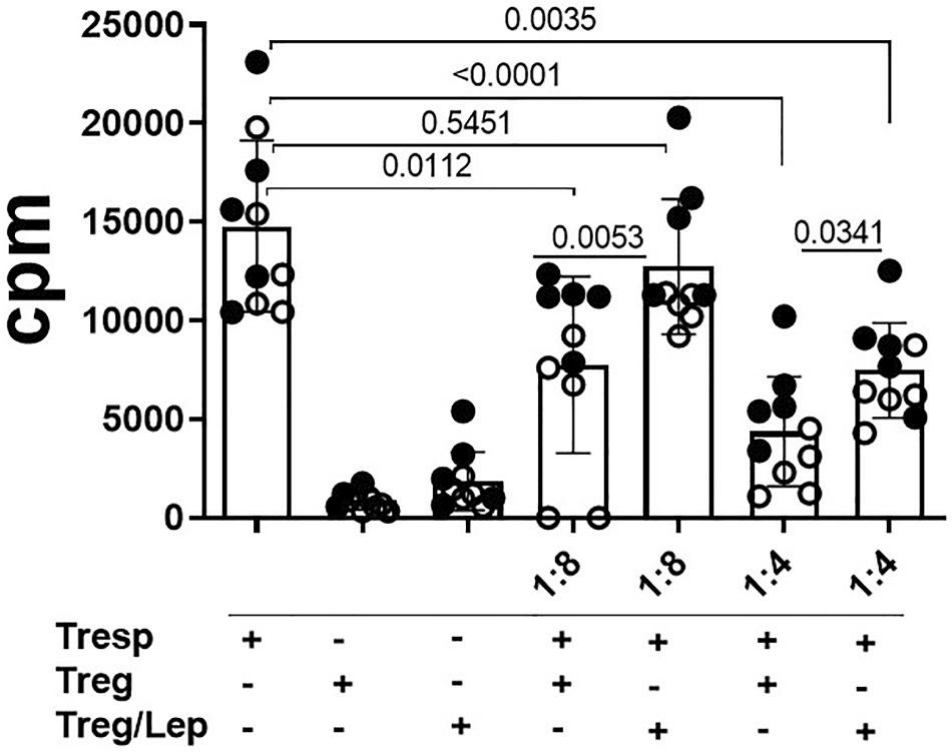
Leptin directly reduces in vitro Treg function. Treg cells (2.5 × 10^4^), previously maintained with medium alone or with leptin, were co‐cultured with Tresp at 1:8 and 1:4 Treg/Tresp ratio in the presence of anti‐CD3/anti‐CD28 beads. After 3 days, cell proliferation was determined through [3 H] thymidine up take. The mean values of cpm were compared using Student's *t* test and the *p* values are indicated in the figure. The effect of leptin on Treg function was investigated in five independent experiments with two donors each (total 10 donors)

Previous findings demonstrated the relationship between the plasma leptin levels and the frequency of different effector and regulatory T cells in AA patients. As demonstrated in Figure 6, higher levels of IL‐5 (Figure 6B), IL‐6 (Figure 6C), IL‐13 (Figure 6E), and IL‐17 (Figure 6F), associated with a lower release of IL‐10 (Figure 6D), were observed in purified CD4^+^ T cells from more obese than leaner AA patients. Interestingly, obesity‐related leptin concentration significantly increased the ability of purified CD4^+^ T cells from lean AA patients to produce IL‐5, IL‐13, IL‐17 and IL‐6, but diminished IL‐10 release. No significant difference was observed with regard to IL‐4 (Figure 6A) and IFN‐γ (data not shown). Although no significant difference was observed for pro‐inflammatory cytokines released by obese‐derived cell cultures in response to leptin, this adipokine reduced IL‐10 levels (Figure 6D). Interestingly, in addition to reducing the proportion of IL‐10‐secreting CD4^+^ T cells, leptin significantly damages the ability of Tregs to inhibit Tresp proliferation (Figure 7) in cell cultures from lean (*n* = 5) and obese (*n* = 5) AA patients.

## 4 DISCUSSION

Classically, AA has been described as an adverse immune event mediated by allergen‐specific Th2 cells, which involves the release of IL‐4, IL‐5 and IL‐13, IgE production and mast cell and eosinophil activation.^5^ These granulocytes release different pro‐inflammatory mediators involved in AA immunopathogenesis, particularly leukotrienes (C4, D4 and E4) and PAF.^5^ In the present study, and in agreement with the literature, a higher proportion of total IL‐4‐producing CD4^+^ T cells was detected in the peripheral blood of AA patients when compared to the group of healthy individuals (control group), mainly among patients with severe AA. Nonetheless, and interestingly, we have found that most IL‐4‐secreting CD4^+^ T‐cells in patients with severe AA were IL‐17 positive and IFN‐γ negative. In addition, a higher proportion of typical Th17 cells was also observed in patients with severe AA. This finding is in line with other studies of asthma that demonstrated the complex immunopathogenesis of the disease, which involves other CD4^+^ T‐cell subtypes in lung disease, such as Th17 and IL‐17^+^IL‐4^+^CD4^+^ T‐cells.^10^, ^12^, ^13^, ^14^, ^15^, ^16^, ^17^ Moreover, in addition to IL‐17, higher levels of IL‐5, IL‐6 and IL‐13 were released by activated CD4^+^ T cells from obese AA patients. Regarding IL‐4, the lack of significance in the levels of this Th2‐related cytokine between the two experimental groups may indicate that only IL‐4‐producing Th17‐like cells are associated with AA severity. Notable association was observed between obesity and expansion of circulating hybrid Th2/Th17 and Th17cells in our AA patients, mainly those with severe forms of the disease. These findings corroborate the hypothesis that obesity is linked to worse clinical outcomes for asthma. This adverse association must be related to chronic state of systemic low‐grade inflammation in which the obese subject is conditioned by a greater production of pro‐inflammatory cytokines, such as IL‐1β, IL‐6 and TNFα, and overproduction of some adipokines, particularly leptin.^27^, ^28^

Elevated leptinemia aggravates allergic diseases.^29^, ^30^ Kalmarzi et al.^32^ demonstrated an inverse correlation between plasma leptin levels and lung function in asthma patients. In the present study, even when paired with body mass index, higher plasma leptin levels were quantified in moderate and severe AA patients when compared with the control group. This phenomenon should be associated with the ability of cytokines related to Th17 (IL‐1β, IL‐6 and IL‐17) and Th2 (IL‐4 and IL‐5) cells in up‐regulating the production of this adipokine.^36^, ^37^, ^38^ Concerning CD4^+^ T cell phenotypes, we have found a positive correlation between plasma leptin levels with the frequency of hybrid Th2/Th17 cells and Th17‐like cells. Other authors have already demonstrated the relationship between IL‐17 and leptin,^38^ however, the correlation between this adipokine and hybrid Th2/Th17 cells is new in the literature, since this double phenotype is still little explored.

Regard to the classic Th2‐like cells (IL‐4^+^IFN‐γ^‐^IL‐17^‐^) no correlation was observed with leptin concentrations and this phenotype. Nonetheless, obesity‐related dose of leptin elevated not only the release of IL‐6 and IL‐17, but also IL‐5 and IL‐13 by CD4^+^ T cells in lean‐derived cell cultures from patients. Ciprandi et al.^39^ demonstrated a direct relationship between leptin levels and IgE titers and eosinophil counts in allergic patients. Therefore, there is a possibility that hyperleptinemia amplifies lung damage during acute exacerbations of asthma by directly up regulating the production of cytokines by Th2/Th17 cell subsets.

Different to Th2 cells, classical Th1 cells are not associated with AA, and, in the present study, the percentage of these cells was dramatically reduced in obese AA patients with severe forms of the disease. In addition, the proportion of this CD4^+^ T cell subset was negatively correlated with plasma leptin levels. Classically, by releasing IFN‐γ, Th1 cells are fundamental in the response against intracellular pathogens, since this cytokine elevates microbicidal power of phagocytes and the cytotoxic function of both NK and CD8^+^ T cells.^40^ Thus, this lower frequency of Th1 cells in our patients should help explain, at least in part, why severe AA elevates susceptibility to infections by intracellular pathogens.^41^

In addition to the involvement of different effector CD4^+^ T cell subtypes, development of AA is also associated with damage in IL‐10 production by CD4^+^T and B cells.^42^, ^43^ Here, both AA severity and obesity negatively affected the frequency of Tregs and Tr1 cells. Moreover, higher plasma leptin levels correlated inversely with those cells. Also, obesity‐related leptin level not only decreased IL‐10 production by CD4^+^ T cells, but also reduced the in vitro suppressive function of these lymphocytes from lean and obese AA patients.

Like IL‐10, a cytokine that plays a potent anti‐inflammatory role, the CD39 molecule has also been described as a marker of Treg cell function.^35^ CD39, in association with CD73, metabolizes the extracellular adenosine triphosphate (ATP) molecule into adenosine monophosphate (AMP), a potent immune inhibitor.^35^ Here, lower frequency of CD39^+^FoxP3^+^CD4^+^ T‐cells was observed in patients with moderate and severe AA. Further, a lower frequency of these cells was found in patients with higher plasma levels of leptin. This finding is new for AA, but a study by Cortez‐Espinosa et al.^44^ observed a negative correlation between the proportion of CD39^+^ Treg cells with IBM in patients suffering from type 2 diabetes.

In addition to T cells, IL‐10‐secreting B (Br1) have been associated with protection against allergic diseases.^42^, ^43^ In the present study, we observed a decrease in the proportion of Br1 cells in patients with moderate and, mainly, severe AA. Among these patients, the lowest proportion of Br1 was seen in obese patients with high plasma leptin levels.

Although preliminary, our findings showed that obesity favors the expansion of circulating Th17‐like cells and hybrid Th2/Th17 phenotype associated with AA severity, together with damage to regulatory CD4^+^ T cell subsets and Br1 cells. Our findings also suggest a role for the hyperleptinemic state in this complex immune imbalance, which might help to explain why weight loss positively affects allergy outcomes.^45^

## AUTHOR CONTRIBUTIONS

Patient monitoring and sample collection by Ulisses C. Linhares and Letícia Delphim. Cleonice A. M. Bento, Sudhir Gupta and Carolina Melo Vollmer designed and wrote the paper. Carolina Melo Vollmer, Aleida S. O. Dias, Lana M. Lopes, Taissa M. Kasahara, Júlio Cesar C. Silva and Hilary Cesário Gonçalves performed the experiments. Carolina Melo Vollmer and Lucas Paulo Lourenço analyzed the data. Cleonice A. M. Bento and Sudhir Gupta contributed with vital reagents. All authors participated in critical revision of the manuscript, provided important intellectual input and approved the final version. All authors read and approved the final manuscript.

## CONFLICT OF INTEREST

All authors declare that there are no conflicts of interest.

## Supporting information

Supplementary Material

Supplementary Material

Supplementary Material
