# Supplementary figures and images for: Leptin favors Th17/Treg cell subsets imbalance associated with allergic asthma severity
Source: Clin Transl Allergy. 2022 Jun 14;12(6):e12153. doi: 10.1002/clt2.12153 (PMC9194742; doi:10.1002/clt2.12153)

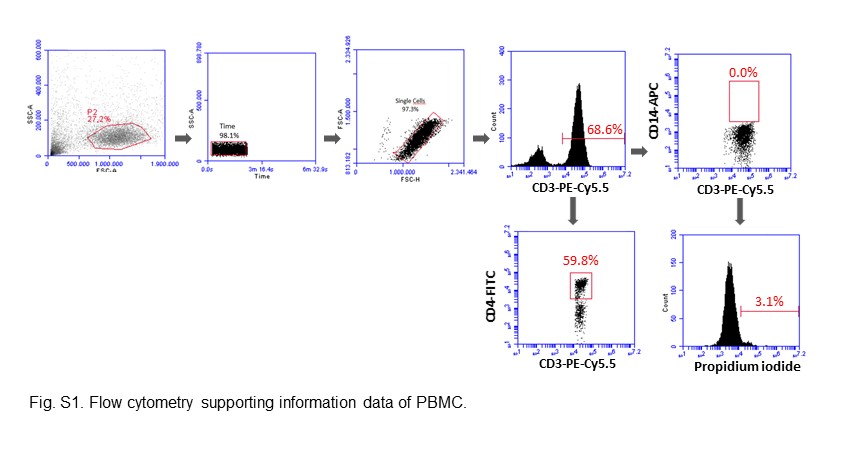

Supplement: Supplementary file 1 — Supplementary Material [file CLT2-12-e12153-s002.jpg]

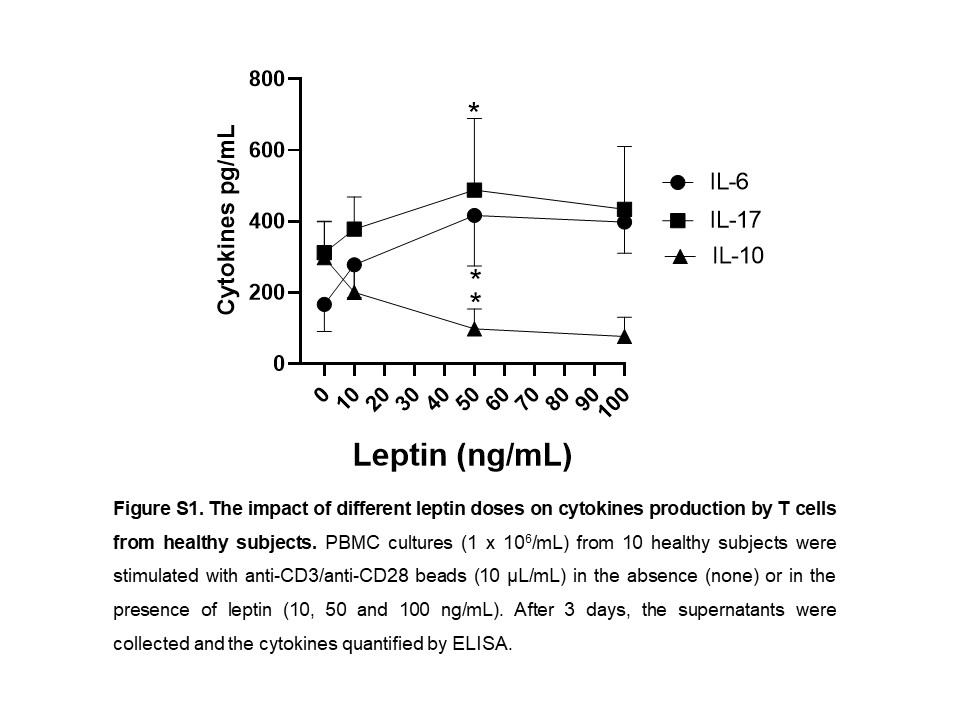

Supplement: Supplementary file 2 — Supplementary Material [file CLT2-12-e12153-s003.jpg]

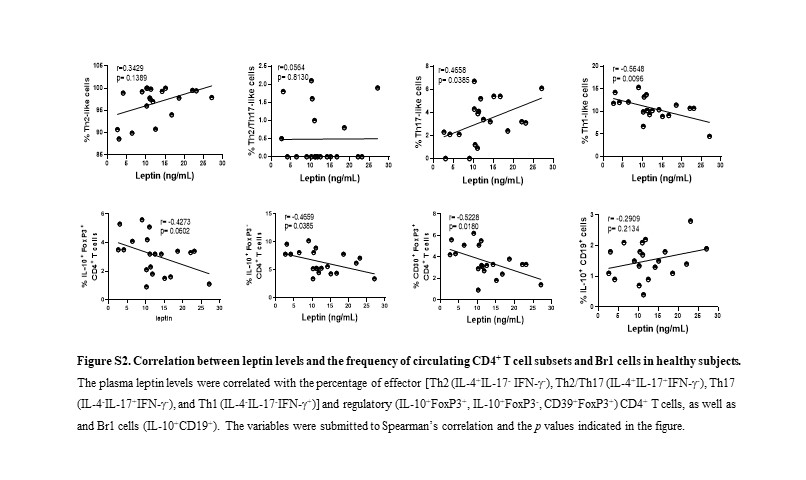

Supplement: Supplementary file 3 — Supplementary Material [file CLT2-12-e12153-s001.jpg]
